# Comorbidities in Patients with Autoimmune Bullous Disorders: Hospital-Based Registry Study

**DOI:** 10.3390/life12040595

**Published:** 2022-04-18

**Authors:** Verónica Sánchez-García, Lorena Pérez-Alcaraz, Isabel Belinchón-Romero, Jose-Manuel Ramos-Rincón

**Affiliations:** 1 Dermatology Department, Alicante Institute of Health and Biomedical Research (ISABIAL), Alicante General University Hospital, 03010 Alicante, Spain; 2 Pharmacy School, Miguel Hernández University, 03202 Elche, Spain; lorena.perez05@alu.umh.es; 3 Clinical Medicine Department, Miguel Hernández University, 03202 Elche, Spain; jose.ramosr@umh.es; 4 Internal Medicine Department, Alicante General University Hospital, 03010 Alicante, Spain

**Keywords:** comorbidity, pemphigus, pemphigoid, vesiculobullous skin diseases, incidence

## Abstract

The incidence of autoimmune bullous disorders has increased over the years, especially in elderly patients with multiple comorbidities, which has stimulated research into their association with other diseases. We performed a retrospective observational study used the Minimum Basic Data Set of hospital discharges to review records of patients admitted to Spanish public hospitals between 2016 and 2019 with a diagnosis of any autoimmune bullous disorder. The objectives were to describe the comorbidity profile and the clinical-epidemiological characteristics of patients with pemphigus and pemphigoid, and analyze the evolution of the incidence of these diseases. The study included 1950 patients with pemphigus and 5424 patients with pemphigoid. Incidence increased from 2016 to 2019. The main comorbidities were hypertension (40.19%) and diabetes mellitus (28.57%). Compared to patients with pemphigoid, those with pemphigus had a higher prevalence of neoplasms, osteoporosis, solid metastases and malignant lymphoma, while the prevalence of hypertension, kidney disease, diabetes, heart failure, dementia, chronic obstructive pulmonary disease and Parkinson’s disease was higher in the pemphigoid group (*p* < 0.05). Therefore, since autoimmune bullous disorders are associated with diverse comorbidities and their incidence has risen in recent years, the establishment of strategies to prevent the main comorbidities in these patients is justified.

## 1. Introduction

Autoimmune bullous disorders (ABDs) are a heterogeneous group of diseases characterized by vesicular-bullous lesions in mucosa and/or skin, caused by the formation of autoantibodies against antigens present in the skin [1]. According to the target structure, ABDs are classified into either the: (i) Pemphigus group, or intraepidermal ABDs, characterized by the formation of autoantibodies against desmogleins, which are intercellular adhesion molecules of keratinocytes [2]; or the (ii) pemphigoid group, or subepidermal ABDs, with autoantibodies against components of the basement membrane zones, which weaken dermal–epidermal cohesion [3,4,5]. The incidence of these diseases has increased over the years, causing a high disease burden and increased healthcare costs [1].

The notable prevalence of ABDs, especially in elderly patients with multiple comorbidities, has been stimulating research into their association with other diseases. The detection of comorbidities in patients with ABDs is therefore important, both to favor optimal therapeutic management and to improve the patient’s final prognosis [6].

Comorbidities that have recently been related to ABDs, especially in patients with bullous pemphigoid, include autoimmune, neuropsychiatric and cardiovascular diseases as well as neoplasms, infections, hypertension and diabetes mellitus [7,8,9].

Notwithstanding this emerging evidence, the literature has scarce data about the relationship between ABDs and some diseases, such as that between pemphigus and neuropsychiatric diseases [10,11]. In addition, most published studies are in patients seen in ambulatory care, whereas few studies analyze the comorbidities of patients with ABD on a hospital basis. In Spain, there is a hospital discharge database, known as the Minimum Basic Data Set (MBDS) [12], which provides information on all hospital admissions and their associated diagnoses, including skin pathologies.

The aim of this study was to describe the clinical-epidemiological characteristics and comorbidity profile in patients with ABDs admitted to hospitals in Spain between 2016 and 2019.

## 2. Materials and Methods

### 2.1. Study Design

This large-scale, retrospective observational study employed the MBDS, an administrative discharge database collecting data on all hospital admissions in the Spanish public healthcare system, and since 2005, in private hospitals as well. This registry collects basic sociodemographic data in hospitalized patients as well as the characteristics of the admission, the primary and secondary diagnoses, procedures performed during hospitalization and circumstances related to hospital discharge. Data were provided by the Health Information Institute of the Ministry of Health, Consumption and Social Welfare [13,14].

We collected data for hospital admissions in patients diagnosed with any subtype of ABD between 2016 and 2019, selecting the following variables: Date of admission, date of discharge, primary and secondary diagnosis of admission, service of admission, type of admission (emergency/elective), type of discharge (home/transfer to another hospital/voluntary discharge/death/transfer to another health center/others), age, sex, nationality and comorbidities.

The “primary diagnosis” refers to the condition considered, upon discharge, to have caused the patient’s admission. Secondary diagnoses (up to 13) are the coexisting diagnoses at or during admission. The type of hospital discharge refers to whether the patient leaves the hospital alive or dead, is transferred to another hospital or is voluntarily discharged.

### 2.2. Study Population

Inclusion criteria were patients admitted to hospitals in Spain from 1 January 2016 to 31 December 2019 with a primary and/or secondary diagnosis of one of the following subtypes of ABD: Pemphigus vulgaris, pemphigus vegetans, pemphigus foliaceus, Brazilian pemphigus or fogo selvagem, pemphigus erythematosus, drug-induced pemphigus, other pemphigus, paraneoplastic pemphigus, unspecified pemphigus, bullous pemphigoid, scarring or mucous membrane pemphigoid, chronic bullous disease of childhood (CBDC), acquired epidermolysis bullosa (AEB) (unspecified/by drugs/other types), other types of pemphigoid and unspecified pemphigoid. Diagnosis was based on the codes in the International Classification of Diseases and Related Health Problems, 10th revision (ICD-10), as detailed in Table S1. Fifteen patients with a dual diagnosis of pemphigus and pemphigoid were excluded from the analysis.

In both the pemphigus and pemphigoid groups, diagnosis of any of the following comorbidities was systematically verified: Acute myocardial infarction, heart failure, peripheral vascular disease, cerebrovascular disease, dementia, chronic obstructive pulmonary disease (COPD), connective tissue disease, ulcer disease, mild liver disease, severe liver disease, type 1 and type 2 diabetes mellitus, hemiplegia, kidney disease, neoplasia, leukemia, malignant lymphoma, solid metastasis, AIDS, arterial hypertension, ischemic heart disease, Parkinson’s disease, anxiety disorder, depressive disorder, schizophrenia, psychosis, bipolar disorder, multiple sclerosis, epilepsy, rheumatoid arthritis, thyroiditis, ulcerative colitis, celiac disease, asthma, osteoporosis, psoriasis and lichen planus. Diagnoses were coded according to the ICD-10 (Table S2).

### 2.3. Statistical Analysis

Sociodemographic and clinical characteristics were compared between the pemphigus and pemphigoid groups using the chi-squared test, the student’s *t* test for the comparison of means in independent groups or analysis of variance (ANOVA, for comparing means in more than two independent groups). Descriptive statistics were expressed as mean (standard deviation, SD) or as absolute and relative quantities (percentages). *p* values of less than 0.05 were considered statistically significant. Statistical analyses were performed with the SPSS program (SPSS: IBM Corp., Armonk, NY, USA), version 25.

Incidence rates for pemphigus and pemphigoid were calculated per 1 million population per year and per 100,000 hospitalizations per year. Population data were obtained through the records of the municipal register of the Spanish National Institute of Statistics [15].

### 2.4. Ethical Aspects

The study protocol was approved by the Clinical Research Ethics Committee of the Alicante General University Hospital (Alicante, Spain) (ref. CEIm: PI2021-119). The use of the MBDS database complies with all relevant Spanish and international legislation regarding data protection and patient privacy, including Organic Law 3/2018 on the Protection of Personal Data and Guarantee of Digital Rights and Directive 95/46/EC on data protection (Law 15/1999). Performance of the study followed Good Clinical Practice and the principles laid out in the Declaration of Helsinki (Fortaleza, 2013).

## 3. Results

### 3.1. Incidence and Trends

**Figure 1:**
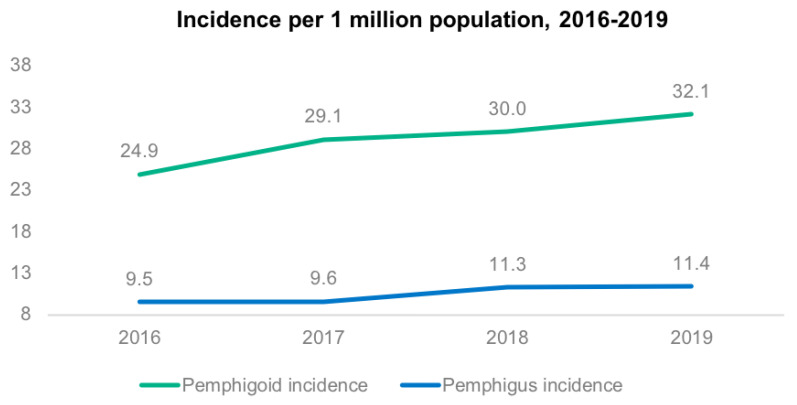
Annual incidence (2016–2019) of pemphigus and pemphigoid per million population.

**Figure 2:**
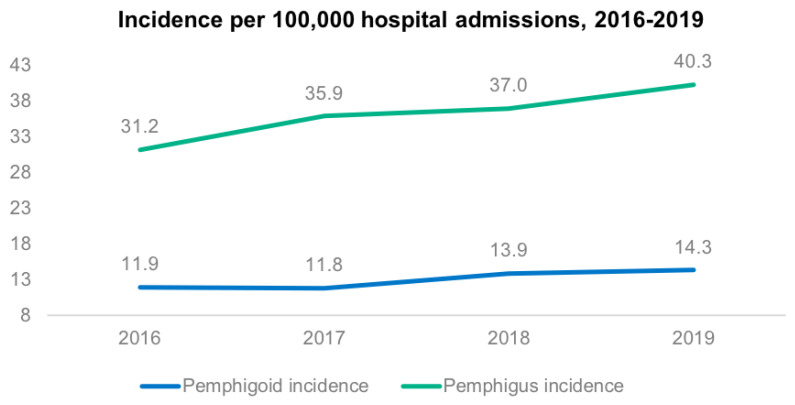
Annual incidence (2016–2019) of pemphigus and pemphigoid per 100,000 hospital admissions.

From 1 January 2016 to 31 December 2019, a total of 15,020,373 admissions were recorded in the MBDS database. Of these, 1950 were diagnosed with some type of pemphigus and 5424 with some type of pemphigoid. Thus, total incidence was, for pemphigus, 13.0 per 100,000 admissions and 10.4 per 1 million population; and for pemphigoid, 36.1 per 100,000 admissions and 29.0 per 1 million population. Incidence increased for both diseases over the study period Figure 1 and Figure 2.

### 3.2. Sociodemographic and Epidemiological Characteristics

A total of 7374 patients were included: 1950 (26.4%) with pemphigus and 5424 (73.6%) with pemphigoid.

**Figure 3:**
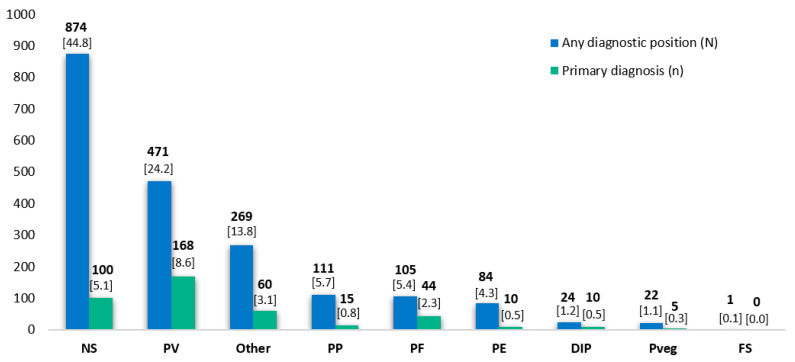
Number of cases by pemphigus subtype and relative importance of diagnosis for the admission (primary versus secondary/other diagnosis). DIP: Drug-induced pemphigus; FS: Fogo selvagem; PE: Pemphigus erythematosus; PF: Pemphigus foliaceus; PP: Paraneoplastic pemphigus; PV: Pemphigus vulgaris; Pveg: Pemphigus vegetans; NS: Pemphigus, not specified. Some patients have >1 pemphigus diagnosis. Number (%) of cases by total of pemphigus.

Patients in the pemphigus group had: Unspecified pemphigus (*n* = 874), pemphigus vulgaris (*n* = 471), other types of pemphigus (*n* = 269), paraneoplastic pemphigus (*n* = 111), pemphigus foliaceus (*n* = 105), pemphigus erythematosus (*n* = 84), drug-induced pemphigus (*n* = 24), pemphigus vegetans (*n* = 22) and fogo selvagem (*n* = 1). Figure 3 shows the frequencies for each within the pemphigus group.

**Figure 4:**
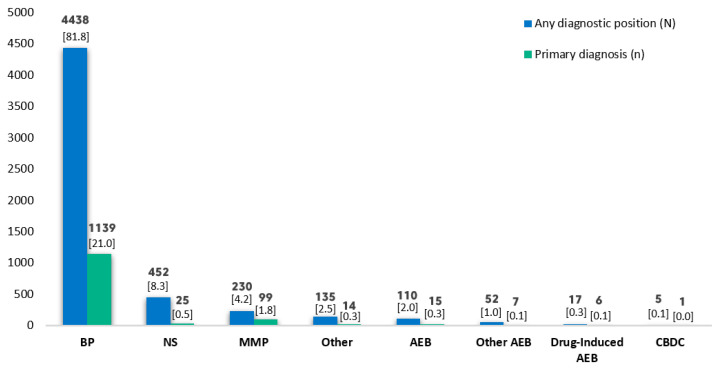
Number of cases by pemphigoid subtype and relative importance of diagnosis for the admission (primary versus secondary/other diagnosis). AEB: Acquired epidermolysis bullosa; BP: Bullous pemphigoid; CBDC: Chronic bullous disease of childhood; MMP: Mucous membrane pemphigoid; NS: Pemphigoid, not specified. Some patients have >1 pemphigoid diagnosis. Number (%) of cases by total of pemphigoid.

Of the 5424 patients with pemphigoid, there were 4438 cases of bullous pemphigoid, 452 of unspecified pemphigoid, 230 of mucous membrane pemphigoid, 135 of other types of pemphigoid, 110 of AEB, 52 of other types of AEB, 17 of drug-induced AEB and five of AEB due to CBDC (Figure 4).

The sociodemographic characteristics of the study participants are detailed in Table 1. Patients with pemphigus were, on average, significantly younger than those with pemphigoid (mean age 71.3 and 80.1 years, respectively; *p* < 0.001). In the pemphigus group there were 947 men (49.9%) and in the pemphigoid group, 2758 (50.8%).

Pemphigus and pemphigoid were the primary diagnoses on admission in 415 and 1309 patients, respectively; the second diagnosis in 170 and 291 patients, the third or subsequent diagnosis in 1365 and 3824.

Regarding the type of admission, patients with pemphigus presented a lower proportion of emergency admissions than those with pemphigoid (80.2% versus 87.7%; *p* < 0.001). The pemphigus group had a slightly higher frequency of admissions to the dermatology service than the pemphigoid group (9.3% and 7.7%; *p* = 0.029). The most frequent reasons for admission in people with pemphigus and pemphigoid according to the major diagnostic categories were, respectively: Diseases of the skin and subcutaneous tissue (23.9% and 27.3%), respiratory diseases (16.2% and 18.9%) and diseases of the circulatory system (11.4% and 12.6%). Mean length of stay was similar (11.1 and 10.6 days, respectively), as was mortality (9.3% and 9.6%, respectively).

### 3.3. Comorbidities

The age-adjusted Charlson Comorbidity Index showed significantly lower scores in the pemphigus compared to the pemphigoid group (4.7 vs. 6.0, *p* < 0.001). For both, the main comorbidities were hypertension and type 2 diabetes mellitus, which were less frequent in patients with pemphigus than with pemphigoid (hypertension: 37.5% vs. 41.2%; diabetes; 24.7% vs. 29.8%; *p* < 0.001). The distribution of comorbidities between the pemphigus and pemphigoid groups is summarized in Table 2.

The comorbidities that were significantly more frequent in patients with pemphigus than with pemphigoid were neoplasms (13.5% vs. 8.5%, *p* < 0.001), osteoporosis (7.4% vs. 5.5%, *p* = 0.003), solid metastases (3.2% vs. 1.9%, *p* < 0.001), malignant lymphoma (2.6% vs. 0.3%, *p* < 0.001) and lichen planus (0.3% vs. 0%, *p* = 0.002). On the other hand, hypertension (37.5% vs. 41.2%, *p* = 0.004), kidney disease (23.2% vs. 37.0%, *p* < 0.001), type 2 diabetes (24.7% vs. 29.8%, *p* < 0.001), heart failure (13% vs. 20.5%, *p* < 0.001), dementia (9.4% vs. 15.3%, *p* < 0.001), COPD (7.3% vs. 10.4%, *p* < 0.001) and Parkinson’s disease (2.4% vs. 3.3%, *p* = 0.043) were more common in the pemphigoid group.

## 4. Discussion

A total of 7374 patients with ABD were admitted to Spanish hospitals from 2016 to 2019. The most frequent subtypes were bullous pemphigoid and pemphigus vulgaris, which is consistent with numerous published studies reporting these as the most frequently diagnosed ABDs [2].

After pemphigus vulgaris, which accounted for 24.2% of the cases of pemphigus in our study, the most prevalent subtypes were paraneoplastic pemphigus (5.7%) and pemphigus foliaceus (5.4%); another 13.8% of cases fell into the category of “other” types. These results are also in line with the literature, which points to these three subtypes as the most common [1,2]. Within the pemphigoid group, after bullous pemphigoid (81.8%), now recognized as the most common subepidermal bullous disease [1], the most frequent subtype was mucous membrane pemphigoid (4.2%). Another 8.3% of cases had “other” types of pemphigoid.

The mean age of patients with pemphigus was 71.3 years, compared to 80.1 years in those with pemphigoid. In the literature, the typical age of onset of pemphigus is 50 to 60 years, although the interval is wide, with cases recorded in both the elderly and children [2]. Pemphigoid usually affects people over 60 years old and its incidence increases exponentially with age [1,16].

Regarding incidence, our results show an upward trend in Spain from 2016 to 2019, for cases of both pemphigus and pemphigoid, per million population and per 100,000 admissions. Overall, pemphigus presented an incidence of 10.4 cases per million pop./year and pemphigoid of 29.0 cases per million pop./year.

When comparing our results with the literature, we observe that the geographical distribution of pemphigus is uneven. While incidence data are limited, it generally ranges from 0.76 to 5 new cases per million population per year. Some ethnic groups, such as Jewish people (16 to 32 new cases per million population per year) and the Japanese (3.5 cases per million population per year) show much higher rates. However, these epidemiological studies have some limitations: Most data were collected 15 to 20 years ago; some registries focus on specific regions rather than the entire population of the country; and the samples are small. Our study overcomes these limitations, using population-based data for virtually all of Spain [1,2,17].

The geographic distribution of pemphigoid also varies in the literature. Its annual incidence has been estimated at 6 to 13 new cases per million population. However, recent studies point to an incidence three times higher. The largest series of patients (*n* = 869) collected in a retrospective historical cohort from the UK, showed higher incidence rates, at 42.8 cases/million population [18]. The rising incidence of pemphigoid in the last two decades has been related to the increased exposure to trigger drugs and higher life expectancy, as well as better diagnosis of non-bullous variants [1,2]. Data from the current literature support the hypothesis that alterations in skin barrier integrity and immune system function associated with aging increase individual susceptibility to developing ABDs in older patients exposed to specific triggers or with comorbidities related to these diseases. Consequently, this has been reported to lead to autoantibody attack, demonstrating the predilection of BP for the elderly population [19].

We also compared the comorbidity profile of patients with pemphigus versus pemphigoid. Several recent studies have investigated comorbidities in patients with ABDs, but most have focused on a single disease group. In our study, the most frequent comorbidities in patients with any ABD were hypertension and diabetes and these were relatively more frequent in the pemphigoid group. These data coincide with Aktas et al’s [20] results, which identified hypertension and diabetes as the main comorbidities linked to ABDs.

Pemphigus has repeatedly been associated with various autoimmune diseases. Parameswaran et al. [21] reported that autoimmune thyroid diseases, rheumatoid arthritis and type 1 diabetes were significantly more frequent in people with pemphigus than in the general population. Other studies also show a higher prevalence of hypertension, diabetes, dyslipidemia, heart disease, asthma, inflammatory bowel disease and osteoporosis in these patients [22,23,24]. In our study, the most commonly diagnosed comorbidities in pemphigus were hypertension, diabetes, kidney disease, neoplasms and ischemic heart disease.

In patients with pemphigoid, the most common comorbidities were hypertension, kidney disease, type 2 diabetes, heart failure and dementia. These diseases, along with COPD and Parkinson’s disease, were significantly more frequent in patients with pemphigoid compared to pemphigus. There are currently many studies investigating the presence of neurological and psychiatric diseases and other comorbidities, such as diabetes and neoplasms, in pemphigoid [6,10,11,20,25,26]. Lee et al. showed that bullous pemphigoid is significantly associated with hypertension, diabetes, chronic kidney disease, basal cell carcinoma and sleep apnea [11]. In a study in Taiwan, there was a higher incidence of hypertension, diabetes, psoriasis, stroke, dementia, Parkinson’s disease and epilepsy in patients with bullous pemphigoid compared to the general population [25,27].

In our study, patients with pemphigoid presented a greater number of comorbidities than patients with pemphigus. Some authors have speculated that the increased comorbidities could be related to the older average age of patients with pemphigoid and their exposure to immunosuppressants, which would facilitate the development of new pathologies or the worsening of pre-existing ones [6].

In recent years, many articles have studied the association between ABDs and neurological diseases. Several have reported that bullous pemphigoid is associated with various neurological and psychiatric diseases, such as dementia, cerebrovascular disease, Parkinson’s, multiple sclerosis and schizophrenia [20,25,28,29,30,31,32,33,34]. However, the relationship between other ABD subtypes and neurological comorbidities has been poorly studied, with no definitive conclusions [10].

We analyzed the presence of different neuropsychiatric diseases in patients with pemphigus and pemphigoid, observing that the pemphigoid group had a significantly higher prevalence of dementia and Parkinson’s disease compared to those with pemphigus. However, there were no statistically significant differences between the two groups in the prevalence of cerebrovascular disease, depression, anxiety, epilepsy, schizophrenia, multiple sclerosis, bipolar disorder or psychosis. More studies are needed to analyze the relationship of these comorbidities in patients with ABDs.

The pathophysiological mechanism underlying the association between neurological diseases and bullous pemphigoid is likewise not fully understood. Some authors have postulated that because the neuronal isoforms of BP180 and BP230 (bullous pemphigoid autoantigens) are expressed in the central nervous system, the neuroinflammation that accompanies neurological diseases would alter the blood-brain barrier and expose these antigens to the immune system. This would lead to a cross-reactive immune response against its cutaneous isoform [35,36]. However, the chronic inflammation caused by bullous pemphigoid could also act as a promoter of neuronal degeneration, making the relationship between bullous pemphigoid and neuropsychiatric diseases bidirectional [6,29].

On the other hand, the association between ABDs and neoplasms remains controversial. Some studies affirm that bullous pemphigoid is the ABD most commonly associated with neoplasms. However, other large case series have failed to find a significant increase in neoplasms in these patients [16,37,38]. The fact that the incidence of both bullous pemphigoid and neoplasms increases with age adds further controversy [7,37]. In patients with pemphigus, neoplasms are usually associated with paraneoplastic pemphigus, although they may also appear less commonly in other pemphigus subtypes. About half of these neoplasms pertain to the lymphoreticular system [2,7,37]. In a German case-control study, pemphigus vulgaris was associated with oropharyngeal, gastrointestinal, colonic and hematologic malignancies, while pemphigus foliaceus was associated with non-melanoma skin cancer [39]. In the patients in our study, the pemphigoid group showed a low prevalence of solid neoplasms, metastases and malignant lymphomas; indeed, the incidence of these diseases was significantly higher in the pemphigus group. Therefore, we concur with the recommendation that pemphigoid patients be screened for neoplasms only in the presence of severe systemic manifestations and/or atypical presentations (e.g., onset in young people).

This study has some limitations. First, the diagnosis of ABDs and comorbidities was based on ICD-10 diagnostic codes rather than a clinical or histological diagnosis. Furthermore, although most ABDs are diagnosed by dermatologists, the coding of these disorders by other specialists could have caused inaccuracies. Second, the study design is retrospective, with data coming from the MBDS. Third, it was impossible to access patients’ medical history to confirm the diagnoses, identify possible causative agents or conduct a post-discharge assessment. Fourth, in many of the included cases, the ABD was not the primary diagnosis of the hospitalization episode; these patients were admitted for other comorbidities and the ABDs were included in the medical record only as personal history. This could explain why the results of our study are somewhat different from those of other registries of patients with ABDs.

On the contrary, this was a large, multicenter study comparing a wide spectrum of comorbidities in patients with pemphigus and pemphigoid, including cardiovascular, autoimmune, neuropsychiatric diseases and neoplasms. Furthermore, despite the aforementioned limitations, the MBDS has demonstrated its usefulness for epidemiological research, covering more than 98% of hospital admissions in Spain [13].

## 5. Conclusions

In conclusion, the present study demonstrates that ABDs are associated with various comorbidities. Compared to patients with pemphigoid, those with pemphigus have a higher frequency of neoplasms, osteoporosis, solid metastases, malignant lymphoma and lichen planus. On the other hand, those with pemphigoid show a relatively higher prevalence of hypertension, kidney disease, diabetes, heart failure, dementia, COPD and Parkinson’s disease. Furthermore, the incidence of ABD continues to rise in Spain, causing a high disease burden. The establishment of prevention strategies for these comorbidities in patients with ABD is justified. In our opinion, it would be cost efficient to establish prevention strategies for cardiovascular risk factors, such as blood pressure measurement or blood glucose controls periodically, with early initiation of specific treatment when values above the cut-off points are detected, thus avoiding the main comorbidities detected in these patients. Likewise, specific treatment for ABDs should be established, either with topical or systemic treatments, with the aim of restoring skin barrier integrity, maintaining cutaneous homeostasis and thus counteracting detrimental systemic effects of skin damage. In addition, more studies are needed to understand whether some diseases are more frequent in patients with ABD due to common pathophysiological pathways or if this is purely coincidental.

## Figures and Tables

**Table 1:** Sociodemographic and epidemiological characteristics of inpatients with pemphigus and pemphigoid in Spain, 2016–2019.

Variable	Pemphigus (N = 1950)	Pemphigoid (N = 5424)	*p* Value
**Age, years, mean (SD)**	71.3 (17.6)	80.1 (12.7)	<0.001 *
**Age groups, n (%)**			
0–17 years	26 (1.3)	24 (0.4)	<0.001 *
18–64 years	509 (26.1)	450 (8.3)	
≥65 years	1415 (72.6)	4950 (91.3)	
**Sex, n (%)**			
Male	947 (49.9)	2758 (50.8)	0.50
Female	976 (50.1)	2666 (49.2)	
**Nationality, n (%)**			
Spain	1455 (74.6)	4187 (77.2)	0.021 *
Other countries	495 (25.4)	1237 (22.8)	
**Diagnostic rank, n (%)**			
Primary diagnosis	415 (21.1)	1309 (24.1)	0.35
Second diagnosis	170 (8.7)	291 (5.4)	
Third diagnosis	158 (8)	339 (6.2)	
4th or other diagnosis	1207 (62.2)	3485 (64.3)	
**Type of admission, n (%)**			
Emergency	1563 (80.2)	4755 (87.7)	<0.001 *
Elective	385 (19.7)	664 (12.3)	
Unknown	2 (0.1)	5 (0.1)	
**Ward of admission, n (%)**			
Dermatology	181 (9.3)	418 (7.7)	0.029 *
Others	1769 (90.7)	5006 (92.3)	
**Reason for admission, by major diagnostic category, n (%)**			
Skin, subcutaneous tissue and breast	467 (23.9)	1480 (27.3)	0.004 *
Respiratory system	315 (16.2)	1027 (18.9)	0.006 *
Circulatory system	222 (11.4)	684 (12.6)	0.16
Kidney and urinary tract	134 (6.9)	469 (8.6)	0.014 *
Digestive system	139 (7.1)	339 (6.3)	0.18
Infectious and parasitic diseases and disorders	108 (5.5)	271 (5.0)	0.35
Musculoskeletal system and connective tissue	96 (4.9)	241 (4.4)	0.38
Others	469 (24.1)	913 (16.9)	ND
**Length of hospital stay (days), mean (SD)**	11.1 (17.8)	10.6 (12.2)	0.16
**Type of discharge, n (%)**			
Home	1619 (83.1)	4491 (82.9)	0.037 *
Transfer to another hospital	61 (3.1)	137 (2.5)	
Voluntary discharge	9 (0.5)	9 (0.2)	
Death	181 (9.3)	521 (9.6)	
Transfer to another (non-hospital) center	53 (2.7)	202 (3.7)	
Other	25 (1.3)	60 (1.1)	
Unknown	2 (0.1)	4 (0.1)	
**Deaths, n (%)**			
Total deaths	181 (9.3)	521 (9.6)	0.68
Primary diagnosis of ABD	14 (3.4)	52 (4)	<0.001 *
2nd or 3rd diagnosis of ABD	17 (5.3)	46 (7.4)	
4th or other diagnosis of ABD	150 (12.4)	423 (12.1)	

* Statistical significance; ABD: Autoimmune bullous disorders; ND: Not done.

**Table 2:** Comorbidities in patients with pemphigus vs. pemphigoid in Spain, 2016–2019.

Comorbidities	Pemphigus (N = 1950) n (%)	Pemphigoid (N = 5424) n (%)	*p* Value
Age-adjusted Charlson index, mean (SD)	4.7 (2.9)	6.01 (2.4)	<0.001 *
Hypertension	731 (37.5)	2233 (41.2)	0.004 *
Type 2 diabetes mellitus	481 (24.7)	1617 (29.8)	<0.001 *
Kidney disease	455 (23.2)	2009 (37.0)	<0.001 *
Neoplasm	263 (13.5)	462 (8.5)	<0.001 *
Heart failure	253 (13)	1114 (20.5)	<0.001 *
Ischemic cardiopathy	243 (12.5)	742 (13.7)	0.18
Dementia	184 (9.4)	852 (15.3)	<0.001 *
Cerebrovascular disease	176 (9)	572 (10.5)	0.057
Osteoporosis	144 (7.4)	298 (5.5)	0.003 *
COPD	142 (7.3)	562 (10.4)	<0.001 *
Mild liver disease	85 (4.4)	260 (4.8)	0.44
Depressive disorder	81 (4.2)	278 (5.1)	0.087
Asthma	68 (3.5)	243 (4.5)	0.061
Solid metastasis	63 (3.2)	104 (1.9)	0.001 *
Peripheral vascular disease	58 (3)	142 (2.6)	0.41
Anxiety disorder	55 (2.8)	134 (2.5)	0.40
Malignant lymphoma	50 (2.6)	18 (0.3)	<0.001 *
Parkinson’s disease	47 (2.4)	181 (3.3)	0.043 *
Epilepsy	47 (2.4)	128 (2.4)	0.90
Connective tissue disease	27 (1.4)	85 (1.6)	0.57
Severe liver disease	27 (1.4)	82 (1.5)	0.69
Psoriasis	27 (1.4)	106 (2)	0.11
Leukemia	26 (1.3)	51 (0.9)	0.14
Hemiplegia	21 (1.1)	41 (0.8)	0.18
Rheumatoid arthritis	17 (0.9)	49 (0.9)	0.90
Ulcerative colitis	13 (0.7)	21 (0.4)	0.12
Schizophrenia	8 (0.4)	32 (0.6)	0.35
Ulcer disease	8 (0.4)	26 (0.5)	0.70
Lichen planus	6 (0.3)	2 (0)	0.002 *
Multiple sclerosis	5 (0.3)	32 (0.6)	0.074
Bipolar disorder	5 (0.3)	12 (0.2)	0.78
Thyroiditis	5 (0.3)	9 (0.2)	0.43
Type 1 diabetes mellitus	4 (0.2)	16 (0.3)	0.51
AIDS	4 (0.2)	5 (0.1)	0.22
Psychosis	2 (0.1)	15 (0.3)	0.17
Celiac disease	0 (0)	0 (0)	-

AIDS: Acquired immunodeficiency syndrome; COPD: Chronic obstructive pulmonary disease; SD: Standard deviation; * Statistical significance.

## Data Availability

Restrictions apply to the availability of these data. Data was obtained from Health Information Institute of the Ministry of Health, Consumption and Social Welfare and are available from the authors with the permission of Health Information Institute of the Ministry of Health, Consumption and Social Welfare.

## Supplementary Materials

The following supporting information can be downloaded at: https://www.mdpi.com/article/10.3390/life12040595/s1, Table S1: Diagnostic codes for pemphigus and pemphigoid, International Classification of Diseases, 10th revision (ICD-10), Table S2: Diagnostic codes for comorbidities studied in relation to pemphigus and pemphigoid, International Classification of Diseases, 10th revision (ICD-10).

## Abbreviations

The following abbreviations are used in this manuscript:

ABDs	Autoimmune bullous disorders
CBDC	Chronic Bullous Disease of Childhood
AEB	Acquired Epidermolysis Bullosa
MBDS	Minimum Basic Data Set
COPD	Chronic Obstructive Pulmonary Disease
ICD-10	International Classification of Diseases and Related Health Problems, 10th revision
