# Supplementary material for: Comorbidities in Patients with Autoimmune Bullous Disorders: Hospital-Based Registry Study
Source: Life (Basel). 2022 Apr 18;12(4):595. doi: 10.3390/life12040595 (PMC9031095; doi:10.3390/life12040595)
Supplement: Supplementary file 1 [file life-12-00595-s001.zip › SUPPLEMENTARY MATERIAL.pdf]

SUPPLEMENTARY MATERIAL

Table S1. Diagnostic codes for pemphigus and pemphigoid, International Classification of Diseases, 10th revision (ICD-10)

ICD-10 codes	Diseases
	**Pemphigus**
**L10.0**	Pemphigus vulgaris
**L10.1**	Pemphigus vegetans
**L10.2**	Pemphigus foliaceus
**10.3**	Brazilian pemphigus or fogo selvagem
**L10.4**	Pemphigus erythematosus
**L10.5**	Drug-induced pemphigus
**L10.81**	Paraneoplastic pemphigus
**L10.89 / L10.8**	Other pemphigus
**L10.9**	Pemphigus, not specified
	**Pemphigoid**
**L12.0**	Bullous pemphigoid
**L12.1**	Scarring or mucous membrane pemphigoid
**L12.2**	Chronic bullous disease of childhood, or linear IgA bullous dermatosis of childhood, or childhood dermatitis herpetiformis
**L12.3 / L12.30**	Acquired epidermolysis bullosa, unspecified
**L12.31**	Epidermolysis bullosa due to drug
**L12.35**	Other acquired epidermolysis bullosa
**L12.8**	Other pemphigoid
**L12.9**	Pemphigoid, unspecified

Table S2. Diagnostic codes for comorbidities studied in relation to pemphigus and pemphigoid, International Classification of Diseases, 10th revision (ICD-10)

Disease	ICD-10 codes
Arterial hypertension	I10, I15, I16
Type 2 diabetes mellitus	E11.0, E11.1, E11.9
Type 2 diabetes mellitus with organ complications	E11.2, E11.3, E11.4, E11.5, E11.6, E11.7
Ischemic cardiopathy	I20-I25
Heart failure	I50, I50.1, I50.2, I50.3, I50.4, I50.5, I50.6, I50.7, I50.8, I50.9
Peripheral vascular disease	I70.90, I73,9, I70.91, I70.92, I70.2
Cerebrovascular disease	I60 - I69
Hemiplegia	G81
Dementia	F01-F04
Parkinson's disease	G20
Multiple sclerosis	G35
Epilepsy	G40, G40.0, G40.00, G40.001, G40.009, G40.01, G40.011, G40.019, G40.1, G40.10, G40.101, G40.109, G40.11, G40.111, G40.119, G40.2, G40.20, G40.201, G40.209, G40.21, G40.211, G40.219, G40.3, G40.30, G40.301, G40.309, G40.31, G40.311, G40.319, G40.A, G40.A0, G40.A01, G40.A09, G40.A1, G40.A11, G40.A19, G40.B, G40.B0, G40.B01, G40.B09, G40.B1, G40.B11, G40.B19, G40.4, G40.40, G40.401, G40.409, G40.41, G40.411, G40.419, G40.5, G40.50, G40.501, G40.509, G40.8, G40.80, G40.801, G40.802, G40.803, G40.804, G40.81, G40.811, G40.812, G40.813, G40.814, G40.82, G40.821, G40.822, G40.823. G40.824, G40.89, G40.9. G40.90, G40.901, G40.909, G40.91, G40.911, G40.919
Anxiety disorder	F41, F41.0, F41.1, F41.3, F41.8, F41.9
Depressive disorder	F32, F32.0, F32.1, F32.2, F32.3, F32.4, F32.5, F32.8, F32.9
Schizophrenia	F20, F20.0, F20.1, F20.2, F20.3, F20.5, F20.8, F20.81, F20.89, F20.9
Psychosis	F22, F23, F28, F29
Bipolar disorder	F31, F31.0, F31.1, F31.10, F31.11, F31.12, F31.13, F31.2, F31.3, F31.30, F31.31, F31.32, F31.4, F31.5, F31.6, F31.60, F31.61, F31.62, F31.63, F31.64, F31.7, F31.70, F31.71, F31.72, F31.73, F31.74, F31.75, F31.76, F31.77, F31.78, F31.8, F31.81, F31.89, F31.9
Rheumatoid arthritis	M06.0-M06.9, M05-M05.9
Thyroiditis	E06, E06.0, E06.1, E06.2, E06.3, E06.4, E06.5, E06.9
Ulcerative colitis	K51, K51.0, K51.00, K51.01, K51.011, K51.012, K51.013, K51.014, K51.018, K51.019, K51.2, K51.20, K51.21, K51.211, K51.212, K51.213, K51.214, K51.218, K51.219, K51.3, K51.30, K51.31, K51.311, K51.312, K51.313, K51.314, K51.318, K51.319, K51.4, K51.40, K51.41, K51.411, K51.412, K51.413, K51.414, K51.418, K51.419, K51.5, K51.50, K51.51, K51.511, K51.512, K51.513, K51.514, K51.518, K51.519, K51.8, K51.80, K51.81, K51.811,

	K51.812, K51.813, K51.814, K51.818, K51.819, K51.9, K51.90, K51.91, K51.911, K51.912, K51.913, K51.914, K51.918, K51.919
Type 1 diabetes mellitus	E10, E10.1, E10.10, E10.11, E10.2, E10.21, E10.21, E10.22, E10.29, E10.3, E10.31, E10.311, E10.319, E10.32, E10.321, E10.3211, E10.3212, E10.3213, E10.3219, E10.329, E10.3291, E10.3292, E10.3293, E10.3299, E10.33, E10.331, E10.3311, E10.3312, E10.3313, E10.3319, E10.339, E10.3391, E10.3392, E10.3393, E10.3399, E10.34, E10.341, E10.3411, E10.3412, E10.3413, E10.3419, E10.349, E10.3491, E10.3492, E10.3493, E10.3499, E10.35, E10.351, E10.3511, E10.3512, E10.3513, E10.3519, E10.352, E10.3521, E10.3522, E10.3523, E10.3529, E10.353, E10.3531, E10.3532, E10.3533, E10.3539, E10.354, E10.3541, E10.3542, E10.3543, E10.3549, E10.355, E10.3551, E10.3552, E10.3553, E10.3559, E10.359, E10.3591, E10.3592, E10.3593, E10.3599, E10.36, E10.37, E10.37X, E10.37X1, E10.37X2, E10.37X3, E10.37X9, E10.39, E10.4, E10.40, E10.41, E10.42, E10.43, E10.44, E10.49, E10.5, E10.51, E10.52, E10.59, E10.6, E10.61, E10.610, E10.618, E10.62, E10.620, E10.621, E10.622, E10.628, E10.63, E10.630, E10.638, E10.64, E10.641, E10.649, E10.65, E10.69, E10.8, E10.9
Celiac disease	K90.0
Asthma	J45, J45.2, J45.20, J45.21, J45.22, J45.3, J45.30, J45.31, J45.32, J45.4, J45.40, J45.41, J45.42, J45.5, J45.50, J45.51, J45.52, J45.9, J45.90, J45.901, J45.902, J45.909, J45.99, J45.990, J45.991, J45.998
Chronic obstructive pulmonary disease	J44
Connective tissue disease	M35
Ulcer disease	K25
Mild liver disease	K70.0, K70.9, K71, K73, K75, K76, K77
Severe liver disease	K70. 2, K70.1, K70.3, K70.4, K74, K74.0-K74.6, K72
Renal pathology	N00-N19
Neoplasia	C00-C76, C80
Leukemia	C91, C92, C93, C94, C95, C96
Malignant lymphoma	C81-C88
Solid metastasis	C77-79
AIDS	B20
Osteoporosis	M81-M81.8, M80-M80.88
Psoriasis	L40, L40.1, L40.2, L40.3, L40.4, L40.5, L40.50, L40.51, L40.52, L40.53, L40.54, L40.59, L40.8, L40.9
Lichen planus	L43, L43.0, L43.1, L43.2, L43.3, L43.8, L43.9
